# Comparison of a Deep Learning Algorithm vs. Humans for Vertebral Heart Scale Measurements in Cats and Dogs Shows a High Degree of Agreement Among Readers

**DOI:** 10.3389/fvets.2021.764570

**Published:** 2021-12-09

**Authors:** Emilie Boissady, Alois De La Comble, Xiajuan Zhu, Jonathan Abbott, Hespel Adrien-Maxence

**Affiliations:** ^1^PicoxIA, Maisons-Alfort, France; ^2^Office of Information Technology, The University of Tennessee, Knoxville, Knoxville, TN, United States; ^3^Department of Small Animal Clinical Sciences, University of Tennessee, Knoxville, Knoxville, TN, United States

**Keywords:** VHS, cardiac, CNN, artificial intelligence, vertebral heart score, dog

## Abstract

Heart disease is a leading cause of death among cats and dogs. Vertebral heart scale (VHS) is one tool to quantify radiographic cardiac enlargement and to predict the occurrence of congestive heart failure. The aim of this study was to evaluate the performance of artificial intelligence (AI) performing VHS measurements when compared with two board-certified specialists. Ground truth consisted of the average of constituent VHS measurements performed by board-certified specialists. Thirty canine and 30 feline thoracic lateral radiographs were evaluated by each operator, using two different methods for determination of the cardiac short axis on dogs' radiographs: the original approach published by Buchanan and the modified approach proposed by the EPIC trial authors, and only Buchanan's method for cats' radiographs. Overall, the VHS calculated by the AI, radiologist, and cardiologist had a high degree of agreement in both canine and feline patients (intraclass correlation coefficient (ICC) = 0.998). In canine patients, when comparing methods used to calculate VHS by specialists, there was also a high degree of agreement (ICC = 0.999). When evaluating specifically the results of the AI VHS vs. the two specialists' readings, the agreement was excellent for both canine (ICC = 0.998) and feline radiographs (ICC = 0.998). Performance of AI trained to locate VHS reference points agreed with manual calculation by specialists in both cats and dogs. Such a computer-aided technique might be an important asset for veterinarians in general practice to limit interobserver variability and obtain more comparable VHS reading over time.

## Introduction

Heart disease is a leading cause of death among aging cats and dogs (1). While echocardiography remains the standard imaging modality for evaluation of cardiac diseases (2), radiology plays an important part in their diagnosis and management. Indeed, as heart disease is frequently associated with enlargement of the cardiac silhouette, this low-cost and widely available exam can be used first as a screening method when a patient is presented with equivocal clinical signs (cough, fatigability, etc.) (3, 4). On plain radiographs, the cardiac silhouette is usually considered to be enlarged when the cardiothoracic ratio (CTR) is >2/3 of the thoracic height and width on the lateral and ventro-dorsal views, respectively (5, 6). On the lateral view, the width is considered enlarged if >2.5–3.5 intercostal spaces (5, 6). However, this semiquantitative method lacks precision, especially in cases of mild-to-moderate cardiomegaly, making the diagnosis arduous for non-specialist veterinarians (7). This is further accentuated by many factors that may interfere with cardiac size evaluation, such as thoracic conformation (for example, barrel-chested dogs physiologically exceed the ranges presented above) or respiratory cycle (6).

To quantitatively evaluate heart size, Buchanan et al. (8) established a method to index heart size to body size, known as the vertebral heart scale (VHS). In the original study published in 1996, the long axis of the heart was measured from the ventral border of the left mainstem bronchus in dogs, and from the intersection of the ventral edge of the trachea and the most ventral apical pulmonary vein in cats to the cardiac apex, while the short-axis measurement was reported to be the maximal dimension perpendicular to the long axis irrespective of anatomical landmarks. An alternative method, using the ventral border of the vena cava as the reference point for the short-axis measurement, has been proposed by Poad et al. (9); however, to the authors' knowledge, there is no study evaluating the impact of this modification on the value of VHS. In addition, Buchanan et al. also suggested a modified method for dogs with an enlarged left atrium. In this case, the long-axis measurement is made from the ventral edge of the elevated left bronchus to the apex of the heart. Each of the cardiac measurements is then reported as a sum using the thoracic vertebrae's length, starting with the fourth one. The main utility of the VHS is to provide the non-specialist with an objective measurement for the identification of cardiomegaly. This method is now recommended by the American College of Veterinary Internal Medicine (ACVIM) for stratification of dogs with mitral valvular disease (10, 11). The VHS is also useful to monitor the patients' heart size over time for screening purposes (12), especially among breed susceptible to acquired heart disease. Moreover, radiological software now usually integrate specialized tools for its measurement on digital radiographs, adding to the convenience of the method. However, the conversion of cardiac axis to vertebral length made by such software is commonly performed using only the vertebral length of T4 to approximate the index, which may lead to overestimation of the VHS.

Recent progress in the field of computer vision gave the medical community access to powerful computer-aided diagnosis (CAD) systems and recently emerged in the field of veterinary medicine (13–15). Simply put, CAD systems can be categorized into classification (the image can have different status and the system has to choose between them, for example, prediction of osteoarthrosis severity on hips radiographs and regression algorithms; the system has to locate specific elements such as anatomical parts or lesions). The latter category includes the identification of keypoints and prediction of their coordinates. Historically, excellent results were described in the field of facial keypoint detection for facial recognition applications (16, 17), but this technology now has various applications in medical imagery, as it allows the automatization of computation of medical imaging indexes based on anatomical landmark detection. Although former machine learning techniques offered an estimation of these indexes, deep learning and convolutional neural networks (CNNs) now equal to overcome human performances (15, 18–21). For instance, deep learning algorithms were developed for CTR calculation in human chest X-rays and were demonstrated to be more reliable, time-efficient, and labor-saving than manual calculation (15). Such concepts can provide non-specialist veterinarians with a quick, precise VHS calculation in which the keypoints used for the index calculation would be accessible to the professional, while eliminating the subjectivity introduced by a human operator.

The aim of this study was to evaluate the performances of an artificial intelligence (AI) algorithm, trained to set the keypoints automatically and independently for VHS measurements in cats and dogs, by comparing its agreement to that of a board-certified cardiologist and a board-certified radiologist. Additionally, the impact of VHS calculation methodology in canine patients on the index value was quantified by comparing the results obtained by the three types of readers using the method originally established by Buchanan and the adapted method described by Hansson.

## Materials and Methods

### Database Construction

Radiographs used for validation of the algorithm were acquired from a referral center institution. All the radiographs were acquired with one of two systems (Philips, DigitalDiagnost or Philips CombiDiagnost R90 MACHINE, Koninklijke Philips, NV, Amsterdam, Netherlands). The Picture Archiving and Communication Software (PACS) of the institution was searched for canine and feline patients with a thoracic study over a 6 months' period (November 2020–March 2021). The studies included at least two orthogonal radiographs. Exclusion criteria were the presence of significant thoracic rotation or presence of radiographic abnormalities affecting cardiac silhouette visualization (pleural effusion, overlying alveolar pattern or masses, etc.). All the studies were exported, anonymized randomized (Google random number generator), and saved in a parent folder as DICOM. Out of the parent folder, 30 canine and 30 feline thoracic studies were selected at random (Google random number generator). A lateral projection (right or left, randomly chosen) for each of those studies was then saved as an 8-bit Joint Photographic Experts Group (JPEG) format before quantitative evaluation.

### Studied Algorithm

The AI algorithm used for VHS calculation in this study was a CNN trained to predict the coordinates of cardiac and vertebral landmarks on lateral radiographs of cats and dogs. More precisely, the CNN, developed using Pytorch library, predicted coordinates of the long and short axes [at the reference point given by the Poad (9), ventral border of the caudal vena cava (CVC)] of the heart, the cranial border of the fourth thoracic vertebra, and seven following intervertebral spaces (targeting middle height of the vertebral bodies). Vertebral points were used to determine vertebral length of cardiac axis.

The architecture of the corresponding CNN was a 121-layer DenseNet custom for leveraging attention. It was pre-trained on predicting cardiomegaly, left atrial dilation, right ventricular dilation, and left ventricular dilation from a dataset described in a previously published work (13). It was then trained with a dropout rate of 5%, a stochastic gradient descent optimizer with a momentum of 0.9, and a weight decay of 1 × 10^−4^. The learning rate was initially set to 0.05 and divided by 10 every five time a new epoch did not improve the validation loss. Early stopping was used to select checkpoint with the lowest mean-square error. Radiographs used for the VHS training were manually annotated by one of the authors (EB). The author manually labeled the landmarks for cardiac width, cardiac height, and individual vertebral length as described above. A standard data augmentation was also used to improve robustness of the CNN: random flip, rotating (±10°), and contrast variation were applied on images.

### Vertebral Heart Scale Measurements Methods (Human Operator)

The VHS was independently calculated by two experienced operators: a board-certified cardiologist (ACVIM-Cardiology) and a board-certified radiologist [American College of Veterinary Radiology (ACVR)]. Measurements were performed using Picoxia proprietary software for simplicity of measurements and to avoid calculation errors. In the corresponding interface, keypoints were automatically pre-positioned. However, to limit the influence of AI-suggested dimensions on human measurements [unconscious bias to attain a similar (or dissimilar) result], each operator had to manually center the measurements cursors before beginning his/her own measurements.

**Figure 1:**
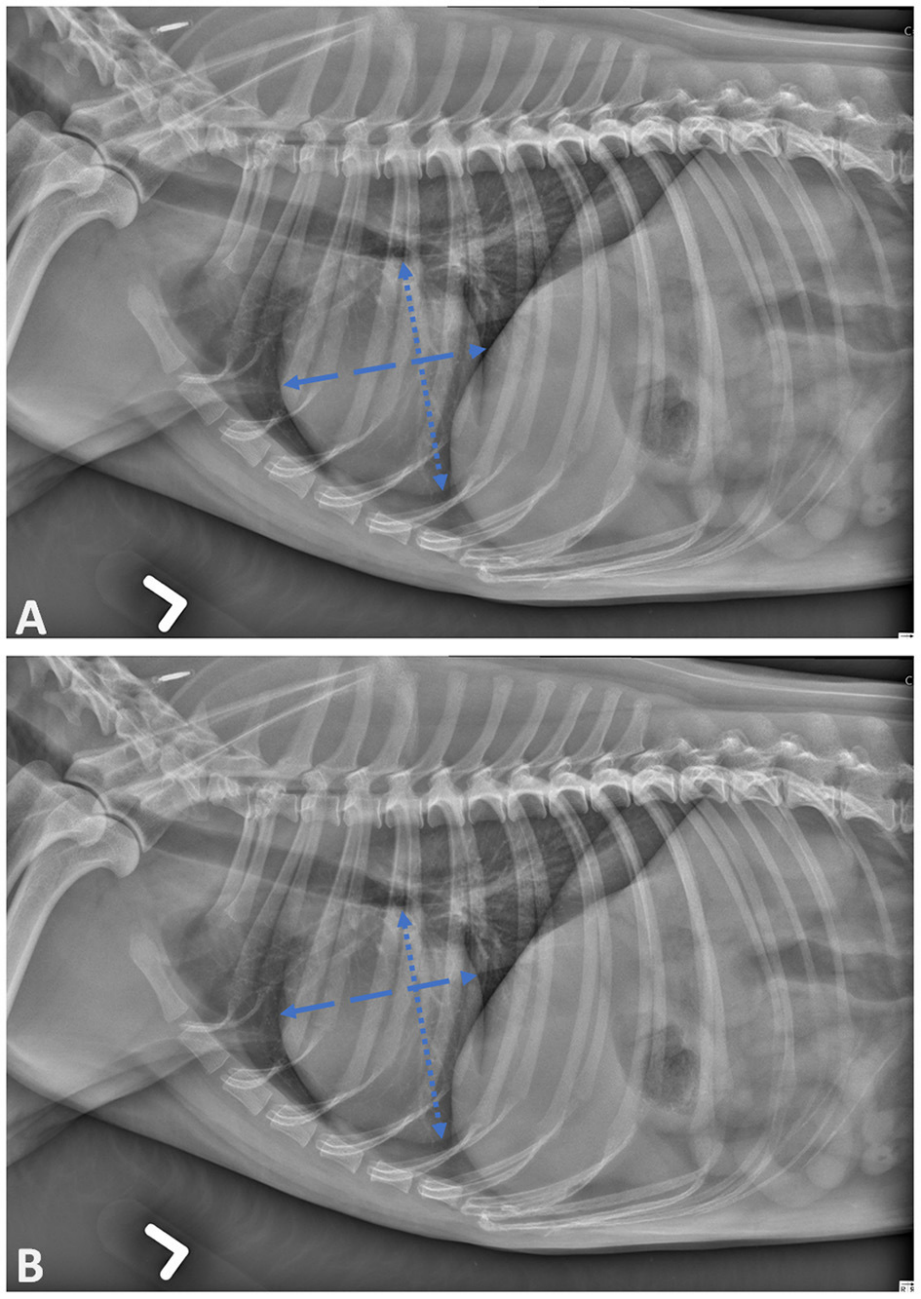
Lateral projection of skeletally mature canine patient demonstrating the cardiac landmarks for vertebral heart scale (VHS) measurements as described by Buchanan **(A)** and Poad **(B)**.

Two measurements methods were applied sequentially on each canine radiograph to evaluate the influence of chosen landmarks on the VHS. The operator had to first use the original approach published by Buchanan (8); the long axis was positioned from the carina to the most distant ventral contour of the cardiac apex and the short axis perpendicular to the long axis, for maximal width (Figure 1A). Then the operator applied the slightly modified approach proposed in the EPIC trial; for the positioning of the short axis, the given consigns were perpendicular to long axis, at the ventral border of the CVC, as illustrated in Figure 1B (9).

In case of an enlarged left atrium, the convention proposed by Buchanan was applied: the long axis was measured from the elevated left bronchus and short axis at the widest level of the heart (8).

**Figure 2:**
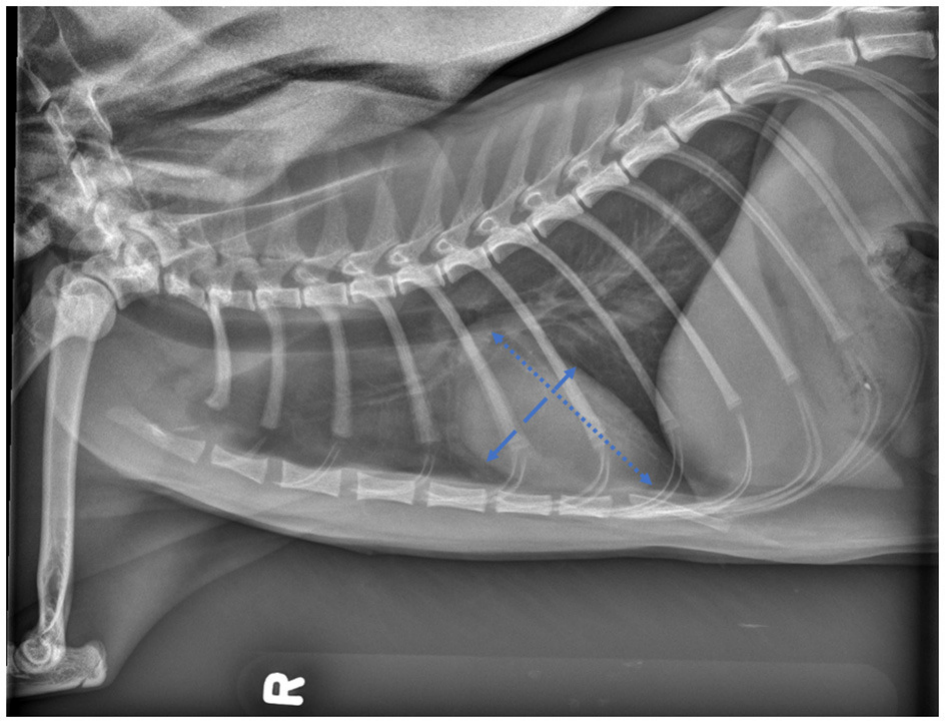
Lateral projection of skeletally mature feline patient demonstrating the cardiac landmarks for vertebral heart scale (VHS) measurements as described by Buchanan.

For feline radiographs, the method proposed by Buchanan was applied (22): the long axis was measured from the intersection of the ventral edge of the trachea and the most ventral apical pulmonary vein to the ventral contour of the cardiac apex, and the short axis was measured perpendicular to the long axis, at the ventral border of the CVC (Figure 2).

### Statistical Analysis

All the statistical analyses were performed by a professional statistician (ZX) using MedCalc software version 20 (MedCalc Software Ltd, Ostend, Belgium; https://www.medcalc.org; 2021).

Intraclass correlation coefficient (ICC) using the absolute agreement between the three types of readers, as well the Bland–Altman plot comparison between the averaged human readers and the AI, was performed. ICC results were qualified as poor if lower than 0.5, moderate if between 0.5 and 0.75, good if between 0.75 and 0.9, and excellent if >0.9. Koo and Li (23) and Jensen and Kjelgaard-Hansen (27) recommended that the acceptable limits of agreement for the Bland–Altman plot were based on inherent imprecision (coefficient of variation (CV), the ratio of the SD to the mean) of both methods in the clinical laboratory. The inherent imprecision (CV) of human readers and AI ranged from 6 to 12%, and the inherent imprecision (CV) of 5% was used as the criteria in this study. In other words, the Bland–Altman plot's limits of agreement intervals were considered within the acceptable range when the bias SD was within the 5% of both methods' mean.

## Results

Overall, the widths, lengths, and VHS calculated by the AI, radiologist, and cardiologist had a high degree of agreement in both canine and feline patients (Table 1). The agreement between the two human specialists was also high (ICC = 0.998).

**Table 1:** Summary table of the ICC for all the measurements performed and their respective categorization.

**ICC**	**ICC among all 3 readers**	**Categorization**
Canine cardiac width (Buchanan method)	0.9813	Excellent
Canine cardiac height	0.9592	Excellent
Canine VHS (Buchanan method)	0.9783	Excellent
Canine cardiac width (EPIC method)	0.9556	Excellent
Canine cardiac VHS (EPIC method)	0.9645	Excellent
Feline cardiac width	0.9308	Excellent
Feline height width	0.9229	Excellent
Feline VHS	0.9469	Excellent

*All the ICCs were categorized as “excellent.”*

*ICC, intraclass correlation coefficient; VHS, vertebral heart scale*.

In canine patients, when comparing the methods used to calculate the VHS by the specialists, there was also a high degree of agreement (ICC = 0.999).

When evaluating specifically the results of the AI VHS vs. the average of the two specialists' readings, using the CVC as the landmark for the short axis positioning in canine patients, the agreement was excellent (the mean of all combined measurements perform by the human operators was 6.43 vs. AI mean of all measurements = 6.28, ICC = 0.998). Bland–Altman analyses Table 2 showed that the mean bias between the CNN and the specialists for VHS was −0.07 vertebra and that the limits of agreement were −0.62 to 0.48. The degree of agreement between AI and human observer was equal in cats and dogs (ICC = 0.998).

**Table 2:** Summary table of the Bland–Altman results.

**Bland–Altman results**	**Bias mean**	**Bias** **SD**	**Limits of agreement**	**Two methods' mean**	**5% (CV) of two methods' mean**
Canine cardiac width (Buchanan method)	−0.06	0.18	−0.41 to 0.29	4.78	0.24
Canine cardiac height	−0.01	0.20	−0.41 to 0.39	5.58	0.28
Canine VHS (Buchanan method)	−0.07	0.28	−0.62 to 0.48	10.36	0.52
Canine cardiac width (EPIC method)	0.14	0.22	−0.30 to 0.58	4.68	0.23
Canine cardiac VHS (EPIC method)	0.14	0.32	−0.50 to 0.77	10.26	0.51
Feline cardiac width	−0.01	0.13	−0.27 to 0.25	3.12	0.16
Feline cardiac height	−0.03	021	−0.45 to 0.39	4.58	0.23
Feline VHS	−0.04	0.29	−0.60 to 0.52	7.70	0.39

*CV, coefficient of variation; VHS, vertebral heart scale*.

All the Bland–Altman plot limits of agreement intervals were within the acceptable range (27).

## Discussion

This study shows that the CNN developed for this project provided a VHS measurement in both and cats, which has a statistically high agreement with the measurements obtained by a cardiologist and a radiologist.

The advantages of using a CNN to calculate the VHS in a general veterinary practice are potentially to limit any interobserver variability and therefore potentially obtain more comparable VHS reading over time for patient's evaluation. The same radiographs can be repeatedly submitted to the CNN, and identical results will be repeated over time. It has been proven by Hansson and al. that while the experience of the observer does not have a significant effect on the VHS measurement, there is a strong interobserver dependency (24). The study compared the results of 16 operators, with evenly distributed experience level, faced with radiographs of 65 Cavalier King Charles Spaniels, and the average difference between the operators was 1.05 ± 0.32 vertebrae. These findings therefore raise concern regarding interobserver reproducibility of the index calculation and highlight the need for standardization methods especially in the context of VHS follow-up in clinics where several practitioners are involved. The use of CNN trained with well-defined landmarks and validated accuracy may help improve practices.

The results of this study also confirm that the CVC is a good landmark to estimate the maximal cardiac width while giving standardized guidelines to the operator, as measurements obtained with the EPIC trial-modified method were nearly identical to those obtained with Buchanan's original method.

The current study has several limitations. First, it was performed on a relatively small sample of 30 canine and 30 feline thoracic radiographs. A larger population would likely have increased the diversity of patients and the likelihood of including dog with congenital vertebral malformation, or cardiac enlargement. It might have been interesting to evaluate the behavior of the CNN on such cases. For further investigations, it would be valuable to conduct a similar study using a population of patients who are known to have evidence of cardiomegaly or atrial dilation and evaluate if for those patients the AI and the specialists' measurements remain in agreement with one another.

As the purpose of this study was to evaluate the agreement between AI and specialists for the VHS, only the VHS measurements (i.e., vertebral axis lengths) were compared. It is unclear if the agreement among all three types of readers only applies to the calculated lengths or if similar landmarks for the measurements were used among the observers for both cardiac and vertebral points. It is possible that AI and human disagreement in locations of the landmarks were compensated when converted to vertebral size.

It would technically be possible, yet challenging, to record the coordinates of each of the reference points on a digital image and compare their values according to the operator responsible for the positioning. To this date, such a study has not been performed, neither using AI nor between human observers. However, the clinical use of such study would potentially be limited, as all references ranges published to date evaluate only the total measurement that the VHS represents and not how it was measured.

Moreover, this study did not evaluate the ability of the algorithm to predict the existence of an underlying cardiac disease or its occurrence and therefore share the same limitations than the VHS (9, 25, 26).

This study was also conducted on radiographs that had been acquired at an academic institution. This might have led to a positive bias in the quality of the radiographs from both a technique and positioning standpoints. It would be valuable to evaluate the performance of the herein developed CNN, on some cases from alternative sources to potentially assess the impact of the radiographs' quality on the results of CNN.

Finally, in the method originally described by Buchanan, the operator must estimate the proportion of the last vertebra (8). In the current study, this was automatically calculated by the computer. This choice may have both a positive impact and a negative impact. On the one hand, this AI-assisted method provides higher accuracy (11), but on the other hand, the clinical relevance of two-decimal numbers to quantify the VHS can be questioned, especially when reference (cutoff values for congestive heart failure diagnosis) was established by manual measurements.

## Conclusion

This study demonstrated that automated measurements of VHS using AI agree with the assessment of board-certified veterinary specialists. Therefore, AI might be useful for the general practitioner evaluating the size of the cardiac silhouette.

## Data Availability Statement

The data analyzed in this study is subject to the following licenses/restrictions: Radiographs used for this study are part of patient's medical record. Requests to access these datasets should be directed to ahespel@utk.edu.

## Ethics Statement

Ethical review and approval was not required for the animal study because Retrospective study evaluating radiographs previously acquired. No acquisitions done for the purpose of this study.

## Author Contributions

All authors listed have made a substantial, direct, and intellectual contribution to the work and approved it for publication.

## Conflict of Interest

The remaining authors declare that the research was conducted in the absence of any commercial or financial relationships that could be construed as a potential conflict of interest. These authors EB and AC developed the CNN PicoxIA.

## Publisher's Note

All claims expressed in this article are solely those of the authors and do not necessarily represent those of their affiliated organizations, or those of the publisher, the editors and the reviewers. Any product that may be evaluated in this article, or claim that may be made by its manufacturer, is not guaranteed or endorsed by the publisher.
